# Engineering strategies to enhance oncolytic viruses in cancer immunotherapy

**DOI:** 10.1038/s41392-022-00951-x

**Published:** 2022-04-06

**Authors:** Yaomei Tian, Daoyuan Xie, Li Yang

**Affiliations:** 1 State Key Laboratory of Biotherapy and Cancer Center/Collaborative Innovation Center for Biotherapy, West China Hospital, Sichuan University, No. 17, Section 3, South Renmin Road, 610041 Chengdu, Sichuan People’s Republic of China; 2 College of Bioengineering, Sichuan University of Science & Engineering, No. 519, Huixing Road, 643000 Zigong, Sichuan People’s Republic of China

**Keywords:** Cancer therapy, Tumour immunology

## Abstract

Oncolytic viruses (OVs) are emerging as potentially useful platforms in treatment methods for patients with tumors. They preferentially target and kill tumor cells, leaving healthy cells unharmed. In addition to direct oncolysis, the essential and attractive aspect of oncolytic virotherapy is based on the intrinsic induction of both innate and adaptive immune responses. To further augment this efficacious response, OVs have been genetically engineered to express immune regulators that enhance or restore antitumor immunity. Recently, combinations of OVs with other immunotherapies, such as immune checkpoint inhibitors (ICIs), chimeric antigen receptors (CARs), antigen-specific T-cell receptors (TCRs) and autologous tumor-infiltrating lymphocytes (TILs), have led to promising progress in cancer treatment. This review summarizes the intrinsic mechanisms of OVs, describes the optimization strategies for using armed OVs to enhance the effects of antitumor immunity and highlights rational combinations of OVs with other immunotherapies in recent preclinical and clinical studies.

## Introduction

Naturally, carcinogenesis proceeds through a multistep process involving the accumulation of genetic and epigenetic aberrations leading to the production of antigens that differ quantitatively or qualitatively from those produced by healthy cells.^1^ These cancer-specific antigens are processed by antigen-presenting cells (APCs), such as dendritic cells (DCs). They first bind to major histocompatibility complex (MHC) molecules and then are presented on the cell APC surface in antigen–MHC complexes. T lymphocytes interact with their cognate T cell receptors (TCRs) to recognize antigen-MHC complexes in lymph nodes. Although antigen stimulation of a TCR is necessary for T-cell activation and proliferation, an additional costimulation signal is needed. CD28, the primary costimulatory molecule on T cells, stimulates the activation of naive T-cells and promotes cytokine secretion. Upon antigen stimulation and costimulation signaling, cytotoxic lymphocytes (CTLs) are primed and trafficked via the circulatory system to the tumor, ultimately eliminating cancer cells. Killing tumor cells requires not only the generation of CTLs but also physical contact between these T cells and cancer cells.^2^

However, the tumor microenvironment (TME) exhibits highly complex heterogeneity and is characterized by acidic conditions, hypoxia, low immunogenicity and suppressed immune cell function.^3,4^ In addition to a dense extracellular matrix (ECM), the cellular components of the TME consist mostly of tumor cells, stem cells (CSCs), endothelial cells (ECs), cancer-associated fibroblasts (CAFs), and tumor-infiltrating immune cells.^5^ Tumor-infiltrating immune cells include macrophages, neutrophils, DCs, myeloid-derived suppressor cells (MDSCs), natural killer (NK) cells, T cells, and B cells.^4^ The immunosuppressive TME is extensively populated with suppressive immune cells such as MDSCs, regulatory T cells (Tregs) and tumor-associated macrophages (TAMs), but CTLs are lacking in the tumor core.^6^ Despite the high infiltration of CTLs in certain types of tumor tissues, immune checkpoint axes (programmed death ligand-1 (PD-1)/PD-L1, etc.) populate the surface of CTLs or tumor cells.^6^ Hence, the immunosuppressive TME poses great challenges to cancer immunotherapy.

Multiple strategies are used to enhance the role of T cells in cancer immunotherapy. Cancer vaccines aim to elicit antigen-specific T-cell cytotoxicity. Adoptive cell therapies are based on autologous tumor-infiltrating lymphocyte (TIL) therapies, chimeric antigen receptor (CAR) T-cell therapies and antigen-specific TCR therapies, which are all aimed at increasing the infusion of tumor-­fighting immune cells. Immune checkpoint inhibitor (ICI) therapies unleash powerful antitumor T cell responses.^7^ These immunotherapies have revolutionized the field of cancer immunotherapy. However, these immunotherapies benefit only a minority of patients for multiple reasons, such as immune system suppression, lack of cytokine variety, poor APC function, few TILs and weak activity of effector T cells.^8^

Viruses have been used as possible agents to treat cancer for more than a century.^9^ With the development of cloning technology, a variety of viruses could be genetically engineered to selectively infect and lyse tumor cells. The increased understanding of viral mechanisms of action, including activating innate and adaptive antitumor immunity and modulating the TME, prospered virotherapy.^10^ Four OVs have been approved for the treatment of various cancers. Despite the approved OVs, a number of OVs that were used as transgene carriers or combined with other immunotherapies were investigated for their antitumor effects in preclinical or clinical studies. In this review, on the basis of the intrinsic mechanism of OVs, we provide a brief overview of each OV and emphasize the role of unarmed or armed OVs in effectively enhancing antitumor immunity in four ways: abrogating immune suppression, producing cytokine variety, enhancing APC function, and proving effector T-cell function.^8,11^ Furthermore, we discuss the rational combinations of OVs with other immunotherapies that have been tested in recent preclinical and clinical studies.

## OVs

**Fig. 1:**
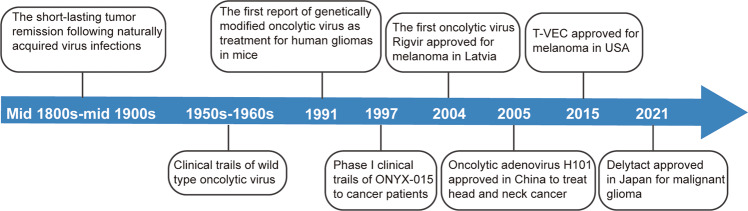
A timeline of important milestones in the development of oncolytic virus as a cancer therapy

Oncolytic virus therapy (OVT) is a novel immunotherapy that uses natural or genetically modified viruses to specifically infect and lyse cancer cells but does not harm normal cells.^12^ Some milestones in the development of OVT are shown in Fig. 1. Historically, the possible use of natural viruses occurred in the early 1900s.^9^ From the early and mid-1900s, patients appeared to have short-lasting tumor remission following naturally acquired virus infections. For example, a 42-year-old woman with acute leukemia presented temporary remission after a presumed influenza infection in 1896.^13^ In the 1950s and 1960s, tests of the in vivo antitumor activity of OVs in patients were capable of being conducted, which benefited from the development of cell and tissue culture systems and the establishment of xenograft murine cancer models.^14^ In 1950, 30 patients with epidermoid cervical carcinomas were treated with 10 different adenovirus serotypes.^15^ Sixty-five percent of the patients formed necrosis and cavitiy in the central portion of cancer tissue. Subsequently, the application of biotechnology technology to genetically engineered viruses accelerated the field of virotherapy.^15^ In 1991, Martuza et al. first reported a thymidine kinase-negative mutant of herpes simplex virus-1 (*dlsptk*) with attenuated neurovirulence, which prolonged survival in glioma-bearing nude mice.^16^

In this period, the modification strategy focused on obtaining tumor selectivity and improving safety. ONYX-015, which was described in 1997, is an attenuated adenovirus with the deletion of E1B55K gene, showing tumor-specific cytolysis and antitumoral efficacy.^17^ The first oncolytic virus, Rigvir, was approved in Latvia in 2004. Rigvir is an unmodified ECHO-7 virus but has been selected for melanoma.^18^ Oncolytic adenovirus H101, with E1B-55KD and partial E3 deleted, became the first approved OV in China in 2005 to treat head and neck cancer.^19^ Engineering OVs with transgenes potentially enhances OV oncolytic activity. T-VEC (IMLYGIC) is a modified form of herpes simplex type-1 virus (HSV-1) that encodes a human granulocyte macrophage colony-stimulating factor (GM-CSF) gene.^20^. T-VEC was approved by the US Food and Drug Administration (FDA) for the treatment of melanoma in October 2015.^21^ The approval of T-VEC has attracted increasing attention to OVT. In 2021, a modified HSV, named Delytact, was approved in Japan for malignant glioma. A multitude of different viruses have been presently exploited as OVs, including adenovirus,^22^ herpes simplex virus,^23^ measles virus,^24^ newcastle disease virus,^25^ reovirus,^26^ vesicular stomatitis virus^27^ and coxsackievirus.^28^

### Adenoviruses

Adenoviruses (AdV) belong to the family of *Adenoviridae*, genus *Mastadenovirus*. They are nonenveloped viruses with double-stranded linear DNA genomes (~30–40 kb) and an icosahedral capsid.^29^ AdV are characterized by hexon, penton-base and fiber proteins, which are responsible for their tropism. Human AdVs are divided into seven different species (A–G) that contain 104 candidate serotypes by April 2021. Serotype 5 adenovirus (Ad5) is the most commonly used viral vector in clinical studies. Ad5 enters the targeted cells via the interaction of fiber knob with coxsackievirus and adenovirus receptors.^30^ Three general strategies have been employed to modify AdV to obtain cancer selectivity. The deletion of the E1A and E1B 55K genes make the AdV selectively replicate in retinoblastoma (pRb)- and p53-mutated tumor cells.^31^ The partial deletion in the E3 region allows AdV to encode immunostimulatory transgenes, which can enhance antitumor immunity.^32^ The Arg-Gly-Asp (RGD) motif was inserted into the HI loop of the AdV fiber protein to improve the infectivity of AdV.^33^

### Herpes simplex virus

HSV, especially HSV type 1 (HSV-1), as an OV, has been tested widely in patients. HSV-1 belongs to the *Alphaherpesvirinae* subfamily of the *Herpesviridae* family. It is ~200 nm in diameter and is a double-stranded DNA virus with a 152 kb genome encoding over 74 distinct genes.^34,35^ The nonessential genes for replication in the large genome could be deleted and replaced with the engineered transgenes, which has no effect on the packaging efficiency of the virus. T-VEC is genetically created through deletion of ICP34.5 and ICP47 and insertion of GM-CSF.^36^ The deletion of ICP34.5, encoding the neurovirulence factor, stops virus replication in neurons but supports virus replication in tumor cells.^37^ Furthermore, in the placement of ICP34.5 T-VEC contains two copies of GM-CSF, which promotes dendritic cell maturation. ICP47 encodes an inhibitor of antigen presentation that blocks MHC class I antigen presentation to CD8^+^ T cells.^38^ Deletion of ICP47 can promote immune responses against tumor cells.^39^

### Vaccinia virus

Vaccinia virus (VV) is an enveloped virus and comprises double-stranded DNA belonging to the genus *Orthopoxvirus* of the *Poxviridae* family.^40^ The genome of VV (70–100 nm in diameter) is approximately 190 kb in length, which allows the insertion and high-level expression of large foreign genes.^41,42^ The deletion of viral thymidine kinase (TK), vaccinia type I IFN-binding protein (B18R) or vaccinia growth factor (VGF) is one of the most common policies to increase the selective replication and lytic capability of VV.^43^ As an oncolytic agent, VV showed a natural selectivity to tumors and a possibility for use with systemic administration.^44^ JX-594 is a Wyeth strain VV-derived OV that lacks the TK gene and is armed with GM-CSF and β-galactosidase.^45^ The deletion of the viral TK gene significantly increased vaccinia specificity to tumors.^46^ The clinical trial JX-594 is discussed below.

### Reovirus

Reovirus (RV) is a nonenveloped, double-stranded RNA (~23.5 kb) virus that belongs to the family *Reoviridae* and has found various hosts in fungi, plants, fish, reptiles, birds and mammals.^47,48^ The double-stranded RNA is structured into 10 segments according to size: large (L1–3), medium (M1–3) and small (S1–4).^49^ Evidence has demonstrated that the Ras signaling pathway is essential for RV replication and the release of virus progeny.^50^ Moreover, RV can induce cell apoptosis through the Ras/RalGEF/p38 pathway.^51^ This makes RV specifically target tumor cells overexpressing Ras. Three different RV serotypes have been identified: type one Lang, type two Jones, and type three Abney and Dearing.^52^ Reolysin (also known as Pelareorep), serotype 3 RV, is the most advanced oncolytic RNA virus in the clinic for cancer therapy and has completed numerous clinical trials as monotherapy or in combination with other therapies.^53^

### Newcastle disease virus

Newcastle disease virus (NDV) is an enveloped virus with negative sense single-stranded RNA from the genus *Avulavirus* of the *Paramyxoviridae* family.^54^ Its diameter is 100–500 nm, and its genome is ~15 kb in length and encodes at least eight proteins (3′-N-P/V/W-M-F-HN-L-5′): nucleocapsid (N), phosphoprotein (P), matrix protein (M), fusion protein (F), hemagglutinin-neuraminidase protein (HN) and large polymerase protein (L)- and two other proteins, V and W.^55^ NDV binds tumor cells through the HN protein, which interacts with sialic acid receptors on the surface of host cells, and then with the activated F protein, the virus and membrane of the host cells fuse with the HN protein. Therefore, the genome of the virus enters the host cytoplasm.^56,57^ The genomes have a large capacity (>5 kb) for the insertion of transgenes, and the insertion site of foreign genes between P/M is recommended. As an oncolytic virus, several clinical studies have demonstrated that NDV has a very high safety profile for cancer patients and shows notable antitumor capacity.^58^

### Measles virus

Measles virus (MeV) is an enveloped virus with negative sense single-stranded RNA from the genus *Morbillivirus* of the *Paramyxoviridae* family. Its diameter is 100–200 nm, and its genome is ~16 kb in length, which includes six genes encoding for eight proteins: six anti-genome arrangements (5′-N-P-M-F-H-L-3′) and two accessory proteins (V and C).^59^ MeV interacts with host cells through three receptors: CD46, signaling lymphocyte-activation molecule (SLAM/CD150) and poliovirus-receptor-like-4 (PVRL4).^60^ SLAM/CD150 is often overexpressed on many hematological malignancies, while CD46 is constitutively overexpressed on many tumor cells, which makes MeV naturally selective for infecting tumor cells.^61^ However, CD46 is also expressed at the basal level in normal cells, so it is not a tumor-selective receptor.^60^ Its favorable safety profile with no dose-limiting toxicities and natural oncotropism makes MeV a promising OV candidate.^62^

### Other oncolytic viruses

Apart from the previously mentioned viruses, several other viruses, such as seneca valley virus,^63^ poliovirus,^64^ vesicular stomatitis virus^65^ and parvovirus^66^ have been developed into oncolytic viruses.

## Mechanism of OV action

**Fig. 2:**
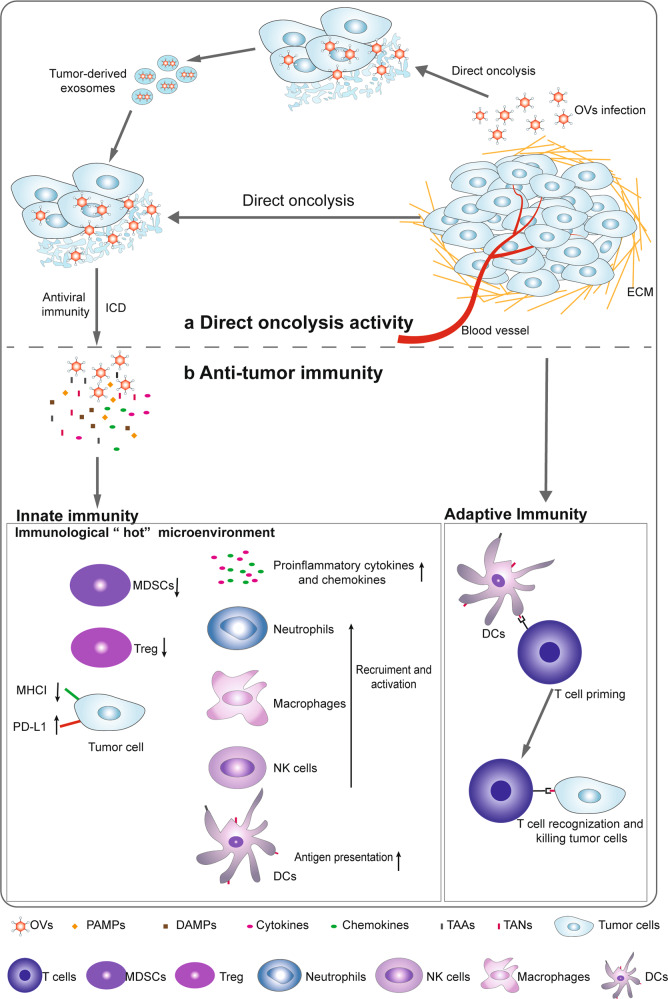
Mechanisms of oncolytic virus (OV) action. **a** Direct oncolysis: new viral particles are released from OV-lysed tumor cells to infect unaffected tumor cells. Moreover, exosomes derived from OV-infected tumors contain OVs and can exhibit high tumor tropism. **b** Antitumor immunity: immunogenic cell death (ICD) induced by OV exposure leads to the release of multiple molecules, including pathogen-associated molecular pattern molecules (PAMPs), damage-associated molecular pattern molecules (DAMPs), tumor-associated antigens (TAAs) and tumor-associated neoantigens (TANs). The identification of PAMPs/DAMPs through pattern recognition receptors (PRRs) in cancer or immune cells triggers the expression of proinflammatory cytokines such as type I interferons (IFNs), interleukin (IL)-1β, IL-6, IL-12, TNF-α, granulocyte macrophage colony-stimulating factor (GM-CSF), and chemokines such as CCL2, CCL3, CCL5 and CXCL10. Chemokines recruit neutrophils and macrophages to infection sites, and these cytokines stimulate the activity of innate immune cells such as NK cells and DCs, which further stimulate the production of IFNs, TNF-α, IL-12, IL-6, and chemokines, resulting in the amplification of the initial innate response and turning immunologically “cold” tumors into “hot” tumors. Type I IFNs increased the levels of MHC class I and II molecules and costimulatory molecules such as CD40, CD80, and CD86 on the surface of DCs. The released TAAs and TANs are processed and ultimately presented on the APC surface in complex with MHC molecules. Multiple cytokines and chemokines contribute to the recruitment and activation of antitumor CD8^+^ T cells and B cells

The direct oncolytic activity of OVs is considered the initial mechanism by which OVs kill cancers.^67^ OVs induce antiviral immunity and antitumor immunity. Antitumor immunity is obviously beneficial for tumor treatment. Based on the premise that the amplification and spread of OVs are limited by the antiviral immune response, host immune responses have been largely assumed to be detrimental to the success of OVs.^68–70^ However, the antiviral immune response has recently been viewed as beneficial in the treatment of tumors for the initial priming of antitumor immunity by OVs.^71^ Here, the direct killing activity and immune response of OVs are described. OVs preferentially target and kill tumor cells without affecting healthy cells. OVs induce innate immunity and turn “cold” tumors into “hot” tumors by facilitating the recruitment of immune cells and activating systemic anticancer adaptive immunity to suppress tumor growth (Fig. 2).

### OVs directly lyse tumor cells

Normal host cells sense viral components and clear viruses by activating signaling pathways. However, abnormalities in the antiviral machinery in tumor cells allow the survival and replication of viruses.^72,73^ OVs are classified as naturally occurring or genetically modified, with the latter targeted to defective antiviral pathways within tumor cells for selectively infecting, replicating and lysing cancer cells, leaving normal cells unharmed.^74,75^ The release of infectious OVs from lysed tumor cells spread to surrounding uninfected tumor cells, resulting in the amplification of their oncolytic activity.^14^

Recently, tumor-derived exosomes secreted after OV infection have been shown to contribute to activated antitumor efficacy. Tumor-derived extracellular vesicles which were obtained from HCT116 tumor-bearing mice infected with oncolytic adenovirus (OAd) OBP-301 contained OBP-301 and exhibited high tumor tropism in orthotopic HCT116 rectal tumors.^76^

### OVs activate innate immunity

Following administration, viral elements known as pathogen-associated molecular patterns (PAMPs), including viral capsids, DNAs, RNAs and proteins, are exposed to the host immune system.^12^ Moreover, OVs can activate various forms of immunogenic cell death (ICD), including immunogenic apoptosis, necroptosis and pyroptosis, by inducing endoplasmic reticulum (ER) stress,^77,78^ leading to the release of hallmark immunostimulatory damage-associated molecular patterns (DAMPs), such as ATP, high mobility group box 1 protein (HMGB1), heat shock protein, ecto-calreticulin and proinflammatory cytokines.^79,80^ These PAMPs and DAMPs are sensed by pattern recognition receptors (PRRs), such as stimulator of IFN genes (STING), Toll-like receptor (TLR) adaptor molecule 1 and TLR3 on immune cells,^81–83^ establishing a proinflammatory microenvironment by stimulating the production of proinflammatory cytokines, such as type I IFNs, interleukin (IL)-1β, IL-6, IL-12, TNF-α, GM-CSF, and chemokines, such as CCL2, CCL3, CCL5, and CXCL10, leading to the transformation of immunologically “cold” tumors into “hot” tumors.^71^ First, locally secreted chemokines, such as CCL3 and CXCL10, recruit the first cell responders, such as neutrophils and macrophages, to the site of infection,^84^ and these cytokines are involved in the induction of effective antitumor responses.^85^ The aggregation of PAMPs with virus-recognizing receptors on NK cells results in the early influx of NK cells. Activated cytotoxic NK cells might kill virus-infected cells by releasing cytolytic components and triggering FAS-FASL signaling.^86^ In addition, activated NK cells express IFN-γ and TNF-α to further contribute to the activation of macrophages, DCs, and T cells.^71^ This NK cell and DC activation further stimulates the production of IFNs, TNF-α, IL-12, IL-6, and chemokines that act in an autocrine and paracrine fashion to amplify the initial innate response.^71,87,88^

### OVs prime antitumor adaptive immunity

The mainstay of adaptive immunity against tumor cells during OV infection is the tumor-specific T-cell response. Successful activation of antigen-specific T-cell responses requires three signals from APCs: antigens presented in the context of an MHC molecule, costimulation, and cytokines. OV-mediated oncolysis of tumor cells initiates the release of tumor-associated antigens and neoantigens (TAAs and TANs, respectively), which are processed by APCs to produce antigen epitopes ultimately presented on the APC surface in complex with MHC molecules. In the cytokine milieu produced after immune and tumor cell exposure to OVs, type I IFNs enhance the expression of MHC class I and II molecules and costimulatory molecules, such as CD40, CD80, and CD86 on the surface of DCs.^89^ Many reports have documented the ability of OVs to induce the activation of MHC class I pathway-related molecules^90,91^ and costimulatory molecules.^92,93^ Notably, multiple cytokines and chemokines produced by OV-infected cells or mature APCs contribute to the recruitment and reactivation of T cells. Once activated, these antitumor CD8^+^ T cells and B cells cause tumor regression and can clear either newly grafted tumors or distant tumors in an OV-independent manner.^94,95^ Therefore, it is being increasingly acknowledged that OVs, including HSV-1,^96^ oncolytic VV, (OVV)^97^ vesicular stomatitis virus (VSV),^98^ MeV,^99^ and OAd,^100^ mainly generate specific and efficacious T-cell immunity to protect against tumors in an antigen-specific manner.

### Effects of OVs on the tumor ECM and vasculature

The ECM, a noncellular compartment, is generated by activated CAFs and comprises up to 60% of a solid tumor mass.^101^ The excessive accumulation of collagenous matrix, proteoglycans, and hyaluronan leads to an impermeable and rigid ECM, forming a shield surrounding tumor cells.^102^ These physical barriers make it difficult for OVs to effectively reach the whole tumor mass.

Ilkow et al. demonstrated that VSV-based therapeutics were enhanced via crosstalk between CAFs and cancer cells.^103^ In contrast, transforming growth factor-beta 1 (TGF-β1) secreted by tumor cells was involved in promoting OV infection of CAFs. In addition, high levels of fibroblast growth factor 2 (FGF2) produced by tumor cells rendered the cells sensitive to viral infection.^103^ Moreover, in addition to killing tumor cells, OAd targeted both glioblastoma cells and glioblastoma‑associated stromal FAP^+^ cells.^104^

OVs have been reported to affect tumor vasculature by infecting and lysing vascular endothelial cells (VECs). Vascular endothelial growth factor (VEGF) suppresses the intrinsic antiviral response and sensitizes tumor vasculature to VV infection by signaling mediated through Erk1/2 and Stat3 and upregulating PRD1-BF1/Blimp1 expression in the tumor vasculature.^105^ Three-dimensional imaging of infected tumors in a murine colon cancer model revealed that VSV replicated in the tumor neovasculature and spread within the tumor mass.^106^ Engineered OVVs were shown to selectively target and disrupt established tumor vasculature, resulting in the destruction of systemic tumors in humans.^107^

## Strategies to enhance OVs’ selective activity

Highly lytic viruses efficiently lyse tumor cells.^108^ There are numerous ways to improve the selective activity of OVs. Early OVs showed a degree of intrinsic oncolytic selectivity that was associated with different gene and protein expression profiles of tumor cells. However, based on the lack of higher specificity, many methods have been used largely to further improve the direct tumor specificity of OVs.^109^ Virulence gene deletion or viral factor modulation is used mainly to maintain OV proliferation and downregulate proapoptotic pathways. T-VEC, a modified HSV-1, was genetically altered through deletion of two nonessential viral genes. Functional deletion of *ICP34.5* and the *ICP47* gene attenuated viral pathogenicity, enhanced tumor-specific cell lysis in a broad range of human tumors and blocked antigen presentation in HSV-infected cells.^110,111^ The approved OAd oncorine was generated by deletion of E1B-55 kDa which binds to the tumor suppressor p53 in normal cells and causes cell cycle progression and viral replication.^112^ Therefore, oncorine does not generally replicate in normal cells but selectively replicates in p53-deficient tumors.^113^ Similarly, to generate oncolytic poxviruses, the viral TK gene is deleted, which increases the selectivity of the virus for rapidly dividing cancerous cells.^114^

Tumor-specific promoters have also been used for the specific delivery of essential genes that induce virus proliferation, particularly OAd proliferation. E1A is an essential gene in adenoviral replication and the first gene expressed upon oncolytic adenoviral infection.^115^ Many tumor-specific promoters that have been utilized to drive E1A expression are strategically applied to improve the specific antitumor activity of OAd, including the human telomerase reverse transcriptase promoter (hTERT), hypoxia-responsive promoter (HRE), prostate-specific antigen promoter (PSA), alpha-fetoprotein promoter (AFP), alpha-lactalbumin promoter (ALA) and mucin1 promoter (DF3/MUC1).^116,117^

Based on mammalian synthetic biology, gene circuits have been creatively engineered to integrate tumor-specific promoters and microRNA (miRNA) inputs for the identification of specific cancer cells.^118^ Huang et al. engineered an innovative sensory switch circuit consisting of a Gal4VP16 activator gene driven by the AFP promoter and two mutually inhibiting repressor genes controlled by miR-142, miR-199a-3p, and miR-142.^119^ In this circuit setup, a high E1A level can be specifically achieved to trigger adenoviral replication in tumor cells.^119^

miRNAs are short small endogenous noncoding RNAs that serve as posttranscriptional regulators of gene expression by interfering with the translation of target mRNAs.^120^ It is now generally accepted that miRNAs are involved in multiple physiological and pathological processes. Dysregulation of miRNAs contributes to tumor progression, invasion, angiogenesis and metastasis in many types of cancers.^121^ miRNAs have been categorized into two classes according to their altered expression in tumor cells: oncogenic miRNA upregulation promotes tumorigenesis by blocking the translation of tumor suppressor protein mRNAs, and tumor suppressor miRNA downregulation generally suppresses the translation of oncoprotein mRNAs.^122^ Hence, elevating tumor suppressor miRNA levels or inhibiting oncogenic miRNA expression is a promising potential therapeutic approach. OV vectors effectively deliver tumor suppressive interfering pre-miRNAs into tumor cells. Specifically, interfering pre-miRNAs are free in the cytoplasm and are cleaved to form mature miRNAs, leading to the inactivation of target mRNAs. OAd carrying the tumor suppressor miRNA-143 (miR-143) induced apoptosis, decreased the expression level of KRAS and reduced tumor growth in HCT116 xenograft cells.^123^ The same antitumor effect of miR-143 was observed in osteosarcoma cells when oncolytic VSV was the carrier.^124^ To enhance oncolytic specificity, Jia et al. inserted miR-34a targets in both the 5′ untranslated region (UTR) and 3′UTR of the virus to obtain double-miR-34a targeting oncolytic coxsackievirus B3, and this engineered virus maintained nearly full oncolytic activity but showed reduced toxicity.^125^ Similarly, oncogenic miRNAs can be used to improve oncolytic safety and specificity. The U_L_9 protein is required for HSV replication, but a dominant-negative mutant inhibits HSV replication by blocking the Ori-binding sites in U_L_9.^126^ miR-21, an oncogenic miRNA, is nearly universally upregulated in cancer cells. Marzulli et al. engineered miR-21-binding sites in the 3′UTR of the dominant-negative U_L_9 gene to enable pre-existing oncogenic miR-21 contact with miR-21-binding sites to restart HSV replication.^126^ In addition, it is thought that cellular miRNAs play important roles via proviral or antiviral effects exerted during the viral life cycle in mammals.^127^ Therefore, the delivery of antiviral miRNAs or the inhibition of proviral miRNA function by OVs is a promising strategy for enhancing oncolytic specificity in tumor cells. miR-222 is a limiting factor for viral propagation, and OAd was engineered with miR-222-binding sites to inhibit high miR-222 expression, leading to cancer cell sensitization to oncolysis.^128^

Although the antiviral immune response has recently been viewed as beneficial in priming antitumor immunity by OVs, antiviral immunity is still considered a hurdle to OV proliferation. Targeting the central mediator of antiviral responses was used to overcome the antiviral response to allow OV proliferation and enhance transgene persistence. Low expression of STAT1, a target gene of IFN signaling of antiviral responses, and its target genes sensitizes melanoma cells to the oncolytic virus EHDV-TAU.^129^ Mutations in the IFNγ–JAK–STAT pathway simultaneously render melanomas susceptible to OV therapy.^130^ CCDC6 has an antiviral influence against the oncolytic alphavirus M1 by regulating IFN-stimulated genes; the epigenetic silencing of CCDC6 sensitizes orthotopic bladder tumors to M1 virus.^131^ Similarly, T-VEC induces ICD in vitro and promotes tumor immunity in low STING-expressing melanoma.^132^ However, Froechlich et al. observed that oncolytic viral replication and cytotoxicity were improved in STING-deficient tumor cells, where oncolytic viruses showed impaired immunogenicity.^133^ Therefore, there is a need to demonstrate the role of antiviral immunity in OV proliferation and the priming of antitumor immunity and propose more strategies to achieve balance, obtaining the maximum effect of OVs.

## Strategies for using armed OVs

### OVs armed with costimulatory molecules enhance APC function

Costimulatory molecules are necessary for the full activation of T cells. In the TME, immunity is suppressed by the lack of costimulatory molecules on the surface of cancer cells. Thus, targeting costimulatory pathways to enhance antitumor immunity seems to be an attractive approach.^134^ Scientists have encoded OVs to express T-cell costimulatory molecules (such as OX40, CD40, intercellular adhesion molecule-1 (ICAM-1), B7-1, lymphocyte function-associated antigen 3 (LFA3), glucocorticoid-induced tumor necrosis family receptor family-related gene (GITR) or 4-1BB) to enhance the antitumor effects of OVs.^135–144^ The latest evidence showed that VALO-D102, a novel AdV encoding CD40L and OX40L, improved tumor growth control and induced robust infiltration of tumor-specific CD8^+^ effector T cells in two mouse models of melanoma. When combined with an anti-PD-1 antibody, VALO-D102 significantly improved tumor suppression compared with either monotherapy alone.^144^ Another OAd, LOAd 703, armed with CD40L and 4-1BBL, promoted the activation of cytotoxic T cells and limited tumor growth in a multiple myeloma xenograft model.^143^ Recent evidence has demonstrated that LOAd 703 can enhance the immunogenic profile by upregulating the costimulatory molecules CD80, CD86, and CD70, MHC molecules, the death receptor Fas and the adhesion molecule ICAM-1.^145^ Currently, two AdVs engineered to express anti-CD40 antibodies or OX40 ligands are being investigated in the clinic: NG-350A and DNX-2440. NG-350A is an OAd vector that expresses a full-length agonist anti-CD40 antibody at the site of viral replication, and DNX-2440 is a replication-competent OAd expressing human OX40 ligand (Table 1).

**Table 1:** Ongoing or completed clinical trials with OVs encoding immunostimulatory transgenes

Virus	Name (Institution)	Transgenes	Tumor type	Reference/identifier	Phase/status
	CG0070 (CG Oncology)	GM-CSF	Bladder cancer	NCT02365818	Phase II Completed
			Nonmuscular invasive bladder cancer	NCT04452591	Phase III Recruiting
	TILT-123 (TILT Biotherapeutics)	TNF-α and IL-2	Solid tumor	NCT04695327	Phase I Recruiting
			Metastatic melanoma	NCT04217473	Phase I Recruiting
Adenovirus	NG-641 (PsiOxus Therapeutics)	Anti-FAP-TAc antibody CXCL9/CXCL10/IFNα	Metastatic cancer Epithelial tumor	NCT04053283	Phase I Recruiting
	ONCOS-102 (Targovax)	GM-CSF	Malignant solid tumor	NCT01598129	Phase I Completed
	NG-350A (PsiOxus Therapeutics)	Anti-CD40 antibody	Metastatic cancer Epithelial tumor	NCT03852511	Phase I Recruiting
	DNX-2440 (DNAtrix)	OX40 ligand	Liver metastases Liver metastasis of Colon cancer Colorectal cancer Breast cancer Gastric cancer Periampullary cancer Melanoma Renal cell cancer Sarcoma Squamous cell carcinoma Gastrointestinal stromal tumors	NCT04714983	Phase I Recruiting
			Glioblastoma	NCT03714334	Phase I Recruiting
	OH2 (Wuhan Binhui Biotechnology)	GM-CSF	Solid tumor Gastrointestinal cancer	NCT03866525	Phase I/II Recruiting
			Pancreatic cancer	NCT04637698	Phase I/II Recruiting
	Talimogene laherparepvec (Amgen)	GM-CSF	Peritoneal surface malignancies	NCT03663712	Phase I Recruiting
			Kaposi sarcoma	NCT04065152	Phase II Recruiting
			Melanoma	NCT04427306	Phase II Recruiting
HSV	VG161 (CNBG-Virogin Biotech)	IL12/15/PDL1B	Advanced malignant solid tumor	NCT04758897	Phase I Recruiting
			Primary liver cancer	NCT04806464	Phase I Recruiting
	M032 (University of Alabama at Birmingham)	IL12	Recurrent glioblastoma Multiforme progressive glioblastoma Multiforme anaplastic astrocytoma or gliosarcoma	NCT02062827	Phase I Recruiting
VV	JX-594 (Jennerex Biotherapeutics Green Cross Corporation)	GM-CSF	Liver cancer	NCT00629759	Phase I Completed
			Melanoma Lung cancer Renal cell carcinoma Squamous cell Carcinoma of the head and neck	NCT00625456	Phase I Completed
			Hepatocellular carcinoma liver cancer (HCC)	NCT01387555	Phase II Completed
			Neuroblastoma Rhabdomyosarcoma Lymphoma Wilm’s tumor Ewing’s sarcoma	NCT01169584	Phase I Completed
			Melanoma	NCT00429312	Phase I/II Completed
	ASP9801 (Astellas Pharma)	IL-7 IL-12	Metastatic cancer Solid tumors Advanced cancer	NCT03954067	Phase I Recruiting
	RGV004 (Second Affiliated Hospital, School of Medicine, Zhejiang University)	Anti-CD19/anti-CD3 bispecific antibody	Relapsed or refractory B-cell lymphoma	NCT04887025	Phase I Not, yet recruiting
VSV	Given IV (Mayo Clinic)	IFN-β NIS	Relapsed or refractory multiple myeloma Acute myeloid leukemia T-cell lymphoma	NCT03017820	Phase I Recruiting

### OVs armed with chemokines recruit antitumor lymphocytes

Chemokines are small secreted proteins that can mediate the migration and positioning of immune cells within various tissues and are involved in the induction and effector phases of immune responses against infections and tumors.^146,147^ Increasing evidence suggests that chemokines play important roles in the TME because of their ability to attract immune cells to tumor lesion sites. Because of this ability, OVs have been armed with chemokines to enhance their antitumor efficacy, especially for turning “cold” tumors into “hot” tumors.^68^ The chemotactic cytokine CCL5 (also known as RANTES), which binds to the receptors CCR1, CCR3, and CCR5 residing on several types of immune cells, including CTLs^148^ and NK cells,^149^ can direct the infiltration of T cells and recruit NK cells via CCR5.^149^ CCL5-armed OVV (vvCCL5)-induced chemotaxis of lymphocyte populations, exerted a great tumor suppressive effect and showed increased levels of TILs when used to simultaneously vaccinate its receptor type-1-polarized dendritic cells.^148^ CCL5-expressing OVV (OV-ffLuc-CCL5) enhanced NK cell accumulation within tumors in vivo.^150^ Additionally, another OAd, Ad-RANTES-E1A, expressed CCL5 in tumors and induced tumor-specific cellular immunity by recruiting myeloid DCs and macrophages to tumor sites.^151–153^ Liu et al. armed an OVV (vvDD) with CXCL11 and found that vvDD-CXCL11 significantly increased CXCL11 protein levels within tumors and recruited CD8^+^ T cells and, to a lesser extent, NK cells to the TME to trigger a systemic antitumor immune response.^154^ Moon et al. also proved that vvDD-CXCL11 was successful in recruiting T cells and augmenting antitumor efficacy.^155^

### OVs armed with cytokines improve antitumor lymphocyte function

Cytokines are soluble proteins that mediate cell-to-cell communication and regulate homeostasis of the immune system.^156,157^ In the TME, cytokines can suppress tumor cell growth through anti-proliferative and proapoptotic activity or recognition by cytotoxic effector cells.^158^ They play very important roles in cancer treatment. Numerous cytokines, including GM-CSF, IL-2, IL-7, IL-12, IL-15, IL-18, IL-21, IL-24, IFN-α, IFN-β, and IFN-γ, can modulate the antitumoral response and have shown antitumor properties in clinical trials and preclinical studies. There are thousands of clinical trials registered with ClinicalTrials.gov that completed recruitment through January 2021 with patients to be treated with cytokines. Among these cytokines, G-CSF, GM-CSF, VEGF, IL-2 and IFN-γ have been the most extensively studied.^159^ However, cytokines generally have short half-lives and act over short distances, limiting their widespread adoption in treatment regimens. Therefore, many OVs have been engineered to express immunostimulatory cytokines in an effort to enhance the antineoplastic immune response.^160^

### GM-SCF

GM-CSF is produced by a variety of cell types, including activated T cells, macrophages, ECs, fibroblasts and cancer cells. It is a potent cytokine that promotes the development and maturation of DCs and the proliferation and activation of T cells, which enhance antitumor immune responses in cancer therapy.^161,162^ GM-CSF is one of the most frequently adopted cytokines for arming OVs. T-VEC encoding GM-CSF was the first OV approved by the US FDA for the treatment of melanoma in October 2015.^20^ Injection with T-VEC induces local and systemic antigen-specific T-cell responses and decreases the number of Tregs, suppressor T cells (Ts), and MDSCs in injected lesions, ultimately leading to an improved durable response rate (DRR) and a long-lasting complete response (CR).^111,163^ Despite T-VEC, GM-CSF is widely used for other types of OVs (HSV,^110^ VV,^164^ VSV,^165^ MV,^166^ AdV,^167,168^ and RV^169^) to enhance its antitumor efficacy. OH2 is derived from wild-type HSV-2 strain HG52, created with the deletion of the ICP34.5 neurovirulence gene and ICP47 gene and expressing the gene encoding human GM-CSF to enhance antitumor immunity.^170^ A single OH2 injection altered the TME with an increase in CD3^+^ and CD8^+^ T cell density and PD-1 expression in patients with metastatic esophageal and rectal cancer.^171^ On August 20, 2021, the US FDA approved OH2 for use in US clinical trials enrolling people with a variety of solid tumors. Studies have proven that JX-594 administered through intravenous infusion continuously spreads infection within tumors but does not harm normal tissues.^172–174^ In phase I/II clinical trials, JX-594 was shown to be well tolerated after intravenous infusion and to induce no dose-limiting toxicities; the maximum tolerated dose was not reached.^172,173,175^ However, JX-594 in combination with sorafenib failed to show a survival benefit in a phase III trial in patients with advanced hepatocellular carcinoma (HCC) without prior systemic therapy (NCT02562755). There are still many issues for JX-594 application to be solved, such as in combination with other immunotherapies.

### Interleukin

Interleukins constitute a class of small-molecule proteins that mediate communication between immune cells and tissue cells, playing important roles in the development and progression of cancers.^176^ Some interleukins can promote tumor growth and metastatic spread (e.g., IL-4, IL-6, and IL-10),^177–179^ while others regulate immunosurveillance and thus tumor control (e.g., IL-2, IL-7 IL-12, IL-15, IL-18, IL-21, IL-23, and IL-24).^180–183^ Therefore, many OVs have been engineered to carry antitumoral ILs.

IL-2 is mainly produced by CD4^+^ T cells and is secreted to a lesser degree by CD8^+^ T cells, B cells, DCs and other innate immune cells.^184,185^ IL-2 can activate both innate and adaptive immunity mainly through effector and regulatory T lymphocytes. IL-2 has been shown to be effective in cancer therapy.^186^ However, the half-life of IL-2 is short (10–85 min in serum),^187^ and therefore, it must be repeatedly administered in short intervals to maintain efficient bioavailability, which limits its clinical use. Recently, scientists have constructed OVs coding IL-2 to ensure that IL-2 can be locally expressed in tumors and to thus enhance OV antitumor activity. Liu et al. demonstrated that IL-2 expressed by OVV was used to treat a variety of murine tumor models and showed no systemic toxicity, and this treatment created an optimal immune microenvironment. Moreover, when combined with an anti-PD-1/PD-L1 antibody, this viral therapy cured most late-stage tumors in mice.^188^ Despite this outcome, OVVs armed with both IL-2 and TNF-α showed even greater effective antitumor efficacy without treatment-related signs of systemic toxicity. This combination treatment enhanced adoptive cell therapy by diminishing the immunosuppressive characteristics of the TME.^189^ IL-2 can be encoded by NDV^190–192^ and HSV,^193^ and it has shown antitumor efficacy against the TME and in the spleen of a late-stage tumor model, as determined by the percentages of activated CD4^+^Foxp3^−^ and CD8^+^IFN-γ^+^ T cells.

IL-12 is known to promote the development of T cells and NK cells and the production of IFN-γ and the T_H_1 response.^194^ All these responses benefit cancer therapy, but in clinical trials, the antitumor efficacy was unsatisfactory.^195,196^ This outcome may have been a result of insufficient IL-12 delivery to the TME or exhaustion of lymphocytes (including T cells, NK cells, TAMs, and/or MDSCs) in the TME. Scientists have used several kinds of OVs armed with IL-12 to solve this problem in many preclinical studies and clinical studies (reviewed by Nguyen et al. ^197^), among which the most commonly used OVs are Ad and HSV. For example, AdV encoding IL-12 (Ad-IL-12) has shown promise as a treatment for cancers (including prostate adenocarcinoma,^198,199^ breast carcinomas,^200^, pancreatic cancer,^201,202^ melanoma,^203^ gliomas^204^ and colorectal carcinomas^205^). In animal tumor models, Ad-IL-12 showed significant antitumor efficacy and prolonged the survival of the animals. The antitumor immune response was mainly mediated by CD8^+^ T cells. Some treated model animals rejected a subsequent rechallenge with the same tumor cells, demonstrating the induction of antitumor immune memory.^195^ In clinical studies, Ad-IL-12 was well tolerated by 21 patients with advanced digestive tumors, and it did not show dose-limiting toxicity.

HSVs armed with IL-12 have also been widely used.^206^ Oncolytic HSV encoding IL-12, oHSV-IL-12, exhibited significant antitumor activity against hepatic tumors and was more effective in rejecting tumor rechallenge. This antitumor efficacy was associated with marked IL-12 and IFN-γ expression, which induced an increase in the number of CD4^+^ and CD8^+^ lymphocytes in the TME.^207^ oHSV-IL-12 elicited local and systemic immune responses, completely preventing the growth of distant untreated lung tumors in mice.^208^ Scientists have also tested the antitumor efficacy of oHSV-IL-12 in ovarian carcinomas,^209^ glioblastoma,^210–212^ neuroblastoma,^213,214^ colorectal cancer^215^ and prostate cancer.^216^ oHSV-IL-12 showed enhanced antitumor efficacy that is mainly mediated by T-cell immune responses. IL-12 was also engineered to be expressed by oncolytic MeV (MeVac FmIL-12), which led to complete remission in 90% of MC38 tumor models.^216^ Enhanced therapeutic efficacy was realized by activation of the systemic antitumor immune response through increased expression of inflammatory cytokines (IFN-γ, TNF-α, and IL-6).^217^

Other viruses, such as VSV,^218^ VV,^219,220^ NDV,^221,222^ and Maraba virus^223^ armed with IL-12, have been used for the rapid improvement in OV antitumor efficacy. To avoid potential systemic toxicity, a number of IL-12 modifications have been explored.^224^ Recently, a double-deleted mutant oncolytic vaccinia virus (vvDD) genetically engineered a membrane-bound IL-12 (vvDD-IL-12-FG) that delivered IL-12 to the tumor bed and tethered IL-12 to cell membranes. vvDD-IL-12-FG inhibited tumor growth and promoted survival without inducing toxic side effects.^225^ The same team also engineered secreted or membrane-bound IL-23, a cytokine in the IL-12 cytokine family, into vvDD to elicit potent antitumor effects by modulating the TME.^226^

IL-15, mainly produced by activated monocytes and macrophages,^227^ primarily promotes the proliferation, activation and cytotoxic functions of CD8^+^ T cells and NK cells.^228^ Studies have reported that IL-15 expressed in the TME may lead to rejection of large tumors by enabling T cells.^229^ Multiple OVs were genetically engineered with IL-15 and have shown promising immunostimulatory and antitumor efficacy.^115,230–232^ IL-15Rα is the IL-15-specific receptor with high affinity. To further enhance IL-15 activity, Kowalsky et al. recently engineered oncolytic VV to express a superagoinst IL-15 (a fusion protein of IL-15 and IL-15Rα) and named it vvDD-IL15-Rα.^232^ As a result, vvDD-IL15-Rα induced strong antitumor activity and prolonged the survival time of tumor-bearing mice. More interestingly, IL-15 promoted the expression of the PD1/PD-L1 axis, which further resulted in a great improvement in the therapeutic outcome via the combination of vvDD-IL15-Rα with PD-1 blockade.

Despite the above common interleukin, a number of other interleukins are armed into OVs to improve antitumor activity, such as IL-7,^220^ IL-36γ, ^233^ IL-21,^234^ IL-24,^235^ and IL-18.^236^ The promising results will advance the clinical applications of IL-armed OVs for tumor treatment as research proceeds.

### Interferons

IFNs, including type I IFNs (IFNα and IFNβ) and type II IFN (IFNγ), comprise a family of cytokines that are recognized as crucial molecules that interfere with viral replication. However, numerous studies have demonstrated that IFNs also play important roles in protecting a host against tumor development through their direct effects on target cells and by activating immune responses.^237^ IFNγ exerts indirect effects on tumor cells via the TME and modulation of the immune response,^238^ and type I IFNs exert direct effects (on cancer cells) and indirect effects (through immune effector cells and vasculature) on tumors.^239^ However, their systemic toxicities and short half-life following administration limit their overall bioavailability.^240^

An engineered OV generated from VSV encoding IFNγ demonstrated greater activation of DCs and induced greater secretion of proinflammatory cytokines than the parental virus and showed pronounced antitumor effects in several murine tumor models.^241^ OAd armed with IFNγ (CNHK300-hIFN-γ) showed antitumor effects through triplex mechanisms, including selective oncolysis, antiangiogenic effects, and immune responses.^242^

Type I IFNs have been frequently inserted into OVs to improve their antitumor efficiency. An IFNα-expressing OAd (RGD-ΔE3-ADP-ham-IFN) showed great therapeutic potential for the treatment of pancreatic cancer in a syngeneic Syrian hamster model.^243^ More recently, an IFNα-expressing OAd (5/3 Cox2 ΔE3 ADP IFN) showed significant tumor growth suppression in an esophageal adenocarcinoma (EAC) xenograft model.^244^ Similarly, multiple types of OVs can be engineered to overexpress IFNβ to improve anticancer efficacy, including VSV,^245–248^ AdV,^249,250^ MeV,^251^ VV,^252^ Sendai virus (SeV),^253^ and NDV.^254^ Despite the effective therapeutic effect of OV-encoding IFN, the potential toxicity should attract attention. Recently, the safety and efficacy of VSV-IFN-NIS, an oncolytic VSV incorporating IFN beta and sodium iodine symporter transgenes, was tested in a phase I clinical trial.^255^ Although a single high-dose intravenous VSV-IFNβ-NIS treatment is safe in heavily pretreated patients with hematologic malignancies, patients still experienced drug adverse events (AEs). A total of 73% (11/15) of patients experienced hematological AEs, particularly lymphopenia (grade 3–4). Nonhematologic AEs of interest were grade 1 (6.7%) and 2 (46.6%) cytokine release syndromes, of which 1 patient required transient norepinephrine support. More strategies and concerns should be provided to achieve the optimal therapeutic effect in patients with OVs or in combination with other immunotherapies but not trigger toxicity (e.g., cytokine storm).

### OVs armed with antigens as cancer vaccines

OV-infected tumor cells resulting from various forms of ICD have been described previously. Their released PAMPs and DAMPs activate innate immunity in the TME, serving as important drivers of tumor cell adjuvanticity.^256,257^ Available TAA and TAN targets derived from OV-infected tumor cells prime antitumor adaptive immunity, which makes antigenicity the other critical advantage of OVs.^257^ Consequently, an oncolytic virus could act as an effective tumor in situ vaccine. An engineered oncolytic herpes virus (OVH) initiates TAA-specific immune responses induced by ICD, which leads to systemic tumor regression in an antigen-targeting therapeutic antibody-dependent manner.^94^ In situ therapeutic cancer vaccination with membrane-tethered IL-2-armed OV (vvDD-mIL2) plus a TLR 9 ligand (CpG) yielded systemic immunization.^258^ Moreover, OVs can also be further armed with tumor antigens to enhance the antitumor immune response. Indeed, in early explorations of this strategy, OV-expressing TAAs (e.g., HPV-16 E7 antigen) were directly used as a vaccine vector to generate an antitumor immune response against TAAs.^259^ Further advancement of this strategy was made through arming OVs to coexpress TAAs and immunomodulatory molecules (e.g., OAd encoding SA-4-1BBL and HPV-16 E7 Antigen^260^), enhancing systemic antitumor immunity. More promising of this approach, investigators have created the heterologous prime-boost cancer vaccination to further expand tumor antigen-specific T cells.^261^ PROSTVAC is a viral but non-OV vector–base cancer vaccine using a prime with vaccinia (PROSTVAC-V) followed by a boost with fowlpox (PROSTVAC-F), each with insertions of four human genes: PSA and three costimulatory molecules LFA-3, B7.1 and ICAM-1.^139^ In a phase II study, PROSTVAC prolonged median overall survival versus placebo in metastatic castration-resistant prostate cancer.^262^ However, PROSTVAC had no effect on median overall survival in metastatic castration-resistant prostate cancer in the phase III study (NCT01322490). The sponsor considered that a few possibilities may account for the findings, including a false-positive signal and/or an imbalance in prognostic factors in phase II and sufficient immune responses or other negative regulatory influences in the TME in phase III.^263^ Then, the sponsor tried the combination therapy in a clinical trial. Bridle *et al*. applied a replication-incompetent adenovirus vector expressing TAAs (e.g., human dopachrome tautomerase (DCT)) to prime and an oncolytic replication-competent rhabdovirus encoding the same TAA as the boosting vaccine. The prime-boost regimens provided outstanding DCT-specific systemic CD4^+^ and CD8^+^ T cell responses,^261^ which were further enhanced by using cyclophosphamide preconditioning.^264^ A recent preclinical study also applied oncolytic Maraba MG1 rhabdovirus encoding MAGE-A3 as a boosting vaccine in primates, and the prime-boost regimen induced an expanded and persistent MAGE-A3-specific CD4^+^ and CD8^+^ T cells. These promising results in preclinical experiments resulted in multiple clinical studies for the treatment of HPV-associated cancers (NCT03618953) and MAGE-A3-positive solid malignancies (NCT02285816, NCT02879760).

### OVs armed with ICIs eliminate immune suppression

Tumor-specific T-cell priming and activation are involved in antigen-specific signaling through TCRs and coactivating signals mediated by cosignaling receptors and costimulatory ligands, but these signaling pathways are disrupted by coinhibitory signaling induced by T cells. Checkpoint receptors such as CTLA4, PD-1, TIGIT, TIM-3, BTLA and CD160.^265^ reside on the T-cell surface. The physical interaction between these checkpoint receptors and their ligands expressed on tumors, APCs and stromal cells leads to coinhibitory signaling, which causes cytotoxic T-cell exhaustion in the tumor environment.^266^ ICI blockade of coinhibitory signaling reverses the exhaustion of CTLs, resulting in the death of tumor cells via restored T-cell functions. Multiple ICIs targeting CTLA4 and PD-1 or PD-L1 have been approved for use in cancer therapy due to their promising long-lasting therapeutic efficacies in many types of cancer.^267^ In addition, ICIs targeting TIGIT,^268,269^ TIM-3^270^ and BTLA^271^ have also demonstrated unprecedented preclinical results and are in clinical development. Scientists have reported that these ICIs cause many immune-related AEs, such as pneumonitis, colitis, and autoimmune diseases.^272^ ICIs can also mediate cardiotoxic effects, which are serious complications that can lead to high mortality.^273,274^ The price of these drugs is very high for patients and health-care systems. Engineering OVs that encode ICIs may be a potential solution to these problems.

A novel recombinant myxoma virus (MYXV) can induce the secretion of the soluble form of PD1 from infected cells. It has been shown to induce and maintain CD8^+^ T cell responses intratumorally. Compared with combination therapy with unmodified myxoma and systemic αPD1 antibodies, MYXV was safer and more effective in a melanoma model.^275^ OAd overexpressing the soluble fusion protein PD-1/CD137L, containing the extracellular domains of PD-1 and CD137L at each terminus, induced tumor-specific and systemic protection against tumors.^276^ MeV-encoding antibodies against CTLA-4 and PD-L1 (MV-αCTLA-4 and MV-αPD-L1) showed high rates of complete tumor remission (>80%) in melanoma xenografts compared with parental MeV.^277^ Kleinpeter et al. demonstrated that OVV-encoded anti-PD1 antibodies (including whole antibodies (mAbs), antigen-binding fragments (Fabs) or single-chain variable fragments (ScFvs)) induced better therapeutic control of tumor growth than either OV or anti-PD1 therapy alone.^278^ In murine models, anti-PD-1 mAb-armed oncolytic HSV showed an enhanced antitumor response, similar to that of unloaded virus combined with anti-PD-1 antibodies, which was superior to that of unloaded virus or anti-PD-1 therapy alone.^279^ T3011 is a genetically modified oncolytic HSV-1 encoding IL-12 and an anti-PD-1 antibody. Locally produced IL-12 induced the synthesis of IFN-γ, enhancing the cytolytic activity of NK cells and CTLs. The anti-PD-1 antibody blocked checkpoint inhibition of T effector cells. The most recent phase I clinical trial reported that T3011 was well tolerated in patients with advanced cutaneous or subcutaneous malignancies.^280^ In another study, a novel oncolytic VV encoding a full mAb against TIGIT showed improved antitumor efficacy and induced long-term tumor-specific immunological memory.^281^ Recently, Lei et al. engineered influenza A virus to express CTLA4-specific scFv to suppress the growth of treated tumors and increase the overall survival of mice.^282^

## OVs combined with immunotherapies

**Fig. 3:**
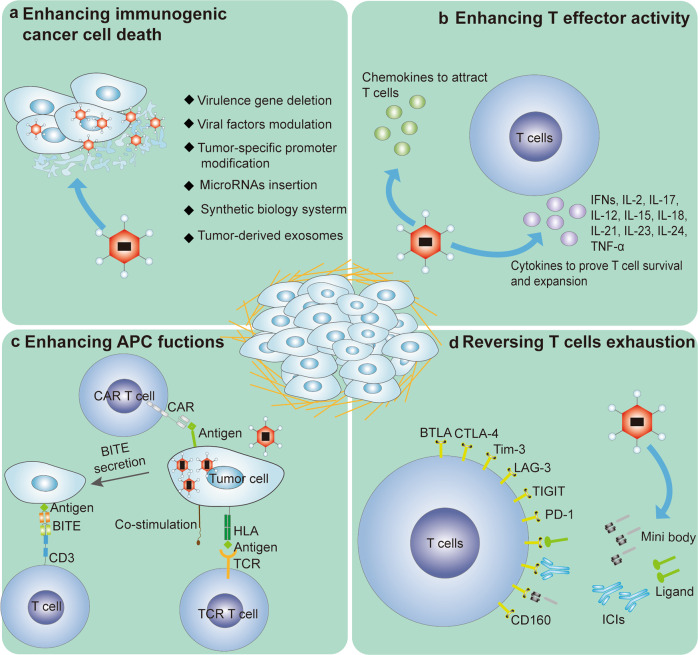
Armed oncolytic virus (OV) enhances antitumor activity. **a** There are numerous means to prove the lytic activity of OVs, some of which might be more immunogenic and prime antiviral adaptive immune responses. **b** The administration of OV-expressing chemokines promotes the secretion of chemokines into the tumor microenvironment (TME), which increases T-cell trafficking to tumors. The secretion of cytokines induced by OVs maintains T-cell survival and expansion. **c** Armed OVs can provide local antigen targets for chimeric antigen receptor T-cell therapy (CAR T) cells or human leukocyte antigen (HLA)/costimulation molecules directed to T-cell receptor (TCR)-T cells. Furthermore, OVs expressing bispecific T-cell engagers (BiTEs) are capable of overcoming antigen heterogeneity and inducing tumor cell death. **d** Immune checkpoint inhibitors (ICIs) or mini bodies and immunosuppressive ligands locally delivered by armed OVs reverse T-cell exhaustion

Unarmed or armed OVs as single agents have demonstrated excellent safety and promising therapeutic effects in tumor treatment. However, monotherapies are unlikely to completely overcome the loss of T-cell function caused by tumor heterogeneity and an immunosuppressive microenvironment. Promising OVs genetically modified with other antitumor agents have achieved tumor eradication in several clinical studies. Recently, the combination of armed OVs with ICIs and adoptive T-cell therapy (ACT) achieved extremely high efficacy by activating multiple antitumor steps, including increasing T-cell trafficking to tumors, supporting T-cell survival and expansion, enhancing APC function and reversing T-cell exhaustion (Fig. 3 and Table 2).

**Table 2:** Ongoing or completed preclinical/clinical studies with OVs and ICIs or ACT therapies

Immunotherapy type	Oncolytic virus	Transgenes	Combination agent/target	Tumor type	Reference/identifier	Phase/status
	Adenovirus (CG0070)	GM-CSF	Pembrolizumab	Nonmuscle invasive bladder cancer	NCT04387461	Phase II Recruiting
	Adenovirus (DNX2401)	None	Pembrolizumab	Glioblastoma and gliosarcoma	NCT02798406	Phase II Completed
	Adenovirus (ONCOS-102)	GM-CSF	Pembrolizumab	Unresectable melanoma	NCT03003676	Phase I Pilot study Completed
	Adenovirus (Telomelysin)	None	Pembrolizumab	Head and beck squamous cell carcinoma (HNSCC)	NCT04685499	Phase II Recruiting
	Vaccinia Virus (BT-001)	CTLA4 Antibody and GM-CSF	Pembrolizumab	Metastatic/advanced solid tumors	NCT04725331	Phase I/II Recruiting
	Vaccinia Virus (TBio-6517)	None	Pembrolizumab	Triple negative breast cancer; Microsatellite instability in colorectal cancer	NCT04301011	Phase I/IIa Recruiting
	Herpes Simplex Virus Type 1 (IMLYGIC)	GM-CSF	Pembrolizumab	Stage IIIB-IVM1d melanoma	NCT04068181	Phase II Active, Not Recruiting
	Herpes Simplex Virus Type 1 (IMLYGIC)	GM-CSF	Pembrolizumab + placebo	Unresectable stage IIIB–IVM1c melanoma (MEL)	NCT02263508	Phase Ib/III Terminated
	Herpes Simplex Virus (ONCR-177)	None	Pembrolizumab	Melanoma; HNSCC; Breast cancer; Triple-negative breast cancer; Colorectal carcinoma; Nonmelanoma skin cancer	NCT04348916	Phase I Recruiting
	Herpes Simplex Virus Type 2 (OH2)	None	Pembrolizumab	Melanoma	NCT04386967	Phase I Recruiting
	Maraba Virus (MG1-MAGEA3) + Ad MAGEA3)	MAGE-A3	Pembrolizumab	Non-small-cell lung carcinoma (NSCLC)	NCT02879760	Phase I/II Completed
Immune checkpoint inhibitors	OVV-01	None	Pembrolizumab or atezolizumab	Advanced solid tumors	NCT04787003	Phase I Recruiting
	Reovirus (REOLYSIN)	None	Pembrolizumab	Pancreatic adenocarcinoma	NCT02620423	Phase Ib Completed
	Coxsackie Virus (CAVATAK)	None	Pembrolizumab	NSCLC	NCT02824965	Phase I/II Active, not recruiting
	Vesicular Stomatitis Virus (VSV-IFNβ-NIS)	IFNβ and the Sodium iodide symporter (NIS)	Pembrolizumab	Refractory NSCLC and HNSCC	NCT03647163	Phase I/II Recruiting
	Vaccinia Viruses (Pexa-Vec)	GM-CSF	Ipilimumab	Metastatic/advanced solid tumors	NCT02977156	Phase I Recruiting
	Herpes Simplex Virus Type 1 (IMLYGIC)	GM-CSF	Ipilimumab + Nivolumab	Triple-negative or estrogen receptor-positive, HER2-negative localized breast cancer	NCT04185311 Phase I	Active, Not Recruiting
	Herpes Simplex Virus Type 1 (HF10)	None	Ipilimumab	Unresectable or metastatic melanoma	NCT03153085	Phase II Completed
	Herpes Simplex Virus Type 1 (HF10)	None	Ipilimumab	Unresectable or metastatic melanoma	NCT02272855	Phase II Completed
	Coxsackievirus (CVA21)	None	Ipilimumab	Uveal melanoma metastases to liver	NCT03408587	Phase I b Completed
	Herpes Simplex Virus Type 1 (RP1)	None	Nivolumab	Advanced and/or refractory solid tumors	NCT03767348	Phase I/II Recruiting
	Herpes Simplex Virus Type 1 (HF10)	None	Nivolumab	Resectable stage IIIB, IIIC, IVM1a melanoma	NCT03259425	Phase II Terminated with Results
	Reovirus (REOLYSIN)	None	Nivolumab + carfilzomib+ dexamethasone	Recurrent plasma cell myeloma	NCT03605719	Phase I Recruiting
	Reovirus (REOLYSIN)	None	Avelumab + paclitaxel	Breast cancer metastatic	NCT04215146	Phase II/III Recruiting
	Poxvirus (JX-594)	GM-CSF and beta-galactosidase	Avelumab + metronomic cyclophosphamide	Sarcoma; Advanced breast cancer	NCT02630368	Phase II Recruiting
	Adenovirus (LOAd703)	4-1BBL+CD40L	Atezolizumab	Pancreatic cancer	NCT02705196	Phase I/IIa Recruiting
	Adenovirus (LOAd703)	4-1BBL+CD40L	Atezolizumab	Malignant melanoma	NCT04123470	Phase I/II Recruiting
	Reovirus (REOLYSIN)	None	Atezolizumab	Early breast cancer	NCT04102618	Early Phase I Recruiting
	Maraba Virus (MG1-E6E7)	Mutant HPV E6 and E7	Atezolizumab	HPV-associated cancers	NCT03618953	Phase I/Ib Active, Not Recruiting
	OVV-01	None	Pembrolizumab or atezolizumab	Advanced solid tumors	NCT04787003	Phase I Recruiting
	Adenovirus (VCN-01)	PH20	Durvalumab	R/M head and neck squamous cell carcinoma		Phase I Recruiting
	Poxvirus (JX-594)	GM-CSF and beta-galactosidase	Durvalumab + tremelimumab	Refractory colorectal cancer	NCT03206073	Phase I/II Active, Not Recruiting
CAR T Cells	Adenovirus (CAdVEC)	None	HER2-CAR T	HER2 positive cancer	NCT03740256	Phase I Recruiting
	Vaccinia Virus (VV.CXCL11)	CXCL11	Mesothelin-CAR T	Lung cancer	^155^	Preclinical Study
	Adenovirus (OAd-TNFa-IL2)	TNF-α and IL-2	Mesothelin-CAR T	Pancreatic ductal adenocarcinoma	^68^	
	Vaccinia Virus (rTTVΔTK-IL21)	IL-21	CD19-CAR T	Lung cancer	^68^	
	Adenovirus (oAD-IL7)	IL-7	B7H3-CAR T	Glioblastoma	^20^	
	Adenovirus (Ad.sTbRFc)	sTGFβRIIFc (targeting TGFβ)	Mesothelin-CAR T	Breast cancer	^20^	
	Adenovirus (OAd-BiTE)	BiTE (targeting EGFR and CD3)	Folate receptor alpha (FR-α)-CAR T	Pancreatic ductal adenocarcinoma	^20^	
	Adenovirus (CAdTrio)	BiTE (targeting CD44v6 and CD3), PD-L1Ab, IL-12p70	HER2-CAR T	Pancreatic ductal adenocarcinoma; Squamous cell carcinoma	^68^	
	Chimeric Orthopoxvirus (OV19t)	CD19	CD19-CAR T	Breast cancer	^68^	
	Vaccinia Virus (mCD19 VV)	mCD19	mCD19-CAR T	Melanoma	^68^	
	Adenovirus (CAd-VECPDL1)	PD-L1 mini-body	HER2-CAR T	Prostate; squamous cell carcinoma	^68^	
CAR NK	Vaccinia Virus (OV-ffLuc-CCL5)	CCL5	CCR5- NK	Colon cancer	^68^	
	Herpes Simplex Virus 1 (OV-IL15C)	IL15/IL15Rα Sushi domain	EGFR-CAR NK	Glioblastoma	^68^	
Adoptive TILs	Poxvirus (vvDD-IL2)	IL-2	TILs	Colon cancer	^68^	

### Combining OVs with ICIs

OVs engineered to encode ICIs are promising beneficial therapies. However, the most commonly used method for treating tumors with ICIs is based on the use of ICI antibodies, such as the approved drugs ipilimumab (anti-CTLA-4), pembrolizumab (anti-PD-1), nivolumab (anti-PD-1), cemiplimab (anti-PD-1), avelumab (anti-PD-L1), and atezolizumab (anti-PD-L1).^283^ Despite the success of these ICIs, only an estimated 12.5% of patients who receive ICI therapy have benefitted.^284^ One of the most commonly recognized reasons for primary resistance to ICI therapy is the absence or low level of PD-L1 on tumor cells.^285^ Initial studies have revealed that primary resistance to ICI therapies was observed when antigen presentation and CD8^+^ T cells were absent in nonimmunogenic tumors.^286^ OVs have been shown to induce a significant increase in PD-L1 levels,^287^ which are beneficial to ICI therapy. Furthermore, OV treatments can turn immunologically “cold” tumors into “hot” tumors, especially through the accumulation of TILs in the tumor tissue.^288^ OVs armed with cytokines broaden the reshaped form of the TME into a proinflammatory microenvironment, rendering tumors more susceptible to ICI therapy.^288^ In a phase Ib study, patients with advanced melanoma exhibited increased CD8^+^ T cells, elevated PD-L1 protein expression, and IFN-γ gene expression in several cell subsets in tumors after IMLYGIC treatment, which benefited from pembrolizumab treatment, resulting in a 62% objective response rate with a 33% CR rate.^289^ Similar results were obtained in a phase II trial evaluating the efficacy and safety of the combination IMLYGIC and ipilimumab in patients with advanced, unresectable melanoma; a higher objective response was observed upon combinatorial treatment compared to ipilimumab alone.^290^ The studies provide a clinical demonstration that the combination of OVs and ICIs could improve therapeutic effects in cancer patients who are resistant to ICIs alone. Based on the evidence, there has been an immense number of preclinical and clinical studies in which both unarmed and armed OVs are combined with ICIs to achieve effective tumor eradication (Table 2).

However, recently, a phase III, randomized, placebo-controlled study of IMLYGIC plus pembrolizumab for unresectable stage IIIB–IVM1c melanoma (MEL) demonstrated that IMLYGIC plus pembrolizumab did not significantly improve progression-free survival or overall survival compared with placebo plus pembrolizumab (NCT02263508). The negative results indicated that the sponsor should consider crucial concerns when selecting the combination of OVs and ICIs, such as tumor subtype and progression, the framework for evaluating changes in tumor size,^291^ the optimal timing of OVs, ICI administration,^292^ etc.

### Combining OVs with CAR T-cell and TCR T-cell therapies

Genetically engineered T-cell immunotherapies have recently achieved inspiring clinical success in the treatment of hematologic malignancies.^293^ The two main approaches to T-cell engineering are the expression of CAR or antigen-specific TCR on T cells, which allows T cells to recognize tumor antigens and ultimately results in the induction of antigen-specific T cell responses.^294^ The most basic framework of CAR involves a genetically incorporated extracellular antigen-specific scFv (the antigen-binding domain), an extracellular hinge region, a transmembrane domain, and an intracellular signaling domain (including CD3ζ and two or more costimulatory domains).^295^ The intracellular signaling domain is designed and enhanced to promote robust cell proliferation, longevity and tumor cytotoxicity in the TME.^293^ TCR-engineered T cells express a recombinant TCR with α and β chains recognizing a TAA to promote antigen-specific immunotherapy.^296^ Recently, CAR T-cell therapy has become a potentially promising treatment for cancer, especially blood cancers. The US FDA has approved CAR T-cell products, Kymriah for treating acute lymphoblastic leukemia,^297^ Yescarta^298^ and Breyanzi^299^ for treating B-cell lymphoma, and Tecartus for treating mantle cell lymphoma.^300^ Clinical trials assessing the effectiveness of TCR T cells showed good outcomes against solid tumors.^301^ However, CAR T cells and TCR T cells have shown suboptimal efficacy against solid tumors.

The efficacy of CAR T and TCR T cells in solid tumors is reduced because of several problems, such as suboptimal trafficking of engineered T cells to tumors, antigen loss or heterogeneity, and poor fit with the tumor immune microenvironment (TIM).^302^ Based on the mechanism, unarmed or armed OVs can overcome barriers to T-cell trafficking to tumors, provide antigens and reverse the immunosuppressive TIM.

### OVs overcome barriers to T-cell trafficking to tumors

The prerequisite for ACT is that CAR T or TCR T cells injected into the bloodstream localize to and infiltrate the tumor core to induce killing of cancer cells. T-cell trafficking to tumors is a multistep process involving adhesion of engineered T cells and local blood vessels, sequentially attaching, rolling, extravasating the vessel and migrating into the tumor core.^303^ However, aberrant chemotactic signaling of chemokine receptors on T cells and chemokines released in the TIM results in inefficient extravasation and recruitment of engineered T cells to the tumor.^2^ Even with proper chemotactic signaling, the tumor vasculature is detrimental to engineered T-cell recruitment because of its high level of disorganization, anergy toward inflammatory stimuli,^303^ and induced endothelial FasL expression that mediates CD8^+^ T cell killing.^2^ Furthermore, Ly6C^lo^ F4/80^hi^ TAMs along the epithelial tumor margins block engineered T-cell infiltration into the tumor.^304^

Positive chemokine–chemokine receptor signaling benefits T-cell trafficking into tumors, including that of the signaling pairs CXCL9,10,11/CXCR3, CXCL16/CXCR6, CCL2/CCR2, CCL3, 4, 5/CCR5, CCL21/CCR7 and CCL27/CCR10.^305^ CAR T cells have been engineered to coexpress CCR2,^306^ CXCR1 or CXCR2,^307,308^ and CCR4^309^ to enhance the ability of T cells to kill tumor cells. TCR T cells armed with CXCR2 markedly improved T-cell homing to a tumor site.^310^ CAR NK cells overexpressing CXCR4 exhibited enhanced migratory capacity compared to conventional CAR NK cells. However, tumor cell- and stromal cell-secreted chemokines that interact with chemokine receptors are essential prerequisites for regulating T-cell infiltration into tumors and influencing therapeutic outcomes in patients. Tumors mostly produce a minute number of chemokines, resulting in inefficient targeting of effectors to tumors.^155^ Despite their intrinsic enhancement of T-cell infiltration, OVs have been genetically engineered to express chemokines such as CCL2,^214^ CCL5,^148^ CCL19,^311^ CXCL11^154^ or CXCL9^312^ to recruit DCs, memory T lymphocytes, CD8^+^ cytotoxic T cells, and CD4^+^ T helper cells into the tumor core, resulting in the expansion of antitumor activity. Based on this idea, chemokine-armed OVs potentially act as powerful enhancers for engineered T-cell immunotherapy. Moon et al. modified CAR T cells to express CXCL11 (CAR/CXCL11) and engineered OVV with CXCL11 (VV.CXCL11).^155^ Although both CAR/CXCL11 and VV. CXCL11 significantly elevated CXCL11 protein levels within tumors; only VV. CXCL11 treatment effectively recruited T cells and augmented antitumor efficacy, which demonstrated the possibility and superiority of OVs as efficient partners in CAR T-cell therapy. OAd engineered to express CCL5 improved the migration of CAR T cells in solid tumors, resulting in increased antitumor effects.^313^ Then, an artificial CCL5–CCR5 axis was activated by inducing CCR5, promoting the differentiation of NK cells in ACT, and OVV was modified with CCL5, inducing the accumulation of NK cells in solid tumors and improving the therapeutic efficacy of NK cells.^150^

### OVs support T-cell survival and expansion

When engineered T cells enter a tumor and confront a hostile TME, the resulting functional exhaustion and insufficient expansion and persistence of the T cells have been identified as major obstacles in ACT.^314^ Cytokines are key contributors to the survival and expansion of T-cell therapies. Therefore, CAR T cells have been genetically engineered to be carriers that deliver cytokines, such as IL-12,^315,316^ IL-15,^317,318^, IL-18,^319^ IL-7,^320^ and IL-23,^321^ into tumors. Additionally, intratumoral production of IL18^322^ or inducible expression of IL-12^323^ with TCR T cells improved the performance of engineered T cells. Compared to CAR T cells and TCR T cells serving as carriers, OVs show superior capacity for delivering cytokines into tumors in ACTs. To date, multiple cytokines, including IFNs, IL-2, IL-17, IL-12, IL-15, IL-18, IL-23, IL-24, and TNF-α, have been introduced into OVs to enhance antitumor immunity.^324^

To date, few preclinical studies using cytokine-armed OVs to improve CAR T-cell therapies have been reported. TNF-α and IL-2 expressed by genetically engineered OAd enhanced the efficacy of mesothelin-CAR T cells in “cold” pancreatic ductal adenocarcinoma. This combination therapy shaped the immunosuppressive TME into a “hot” TME by increasing T-cell recruitment, enhancing T-cell function, driving macrophage polarization into the M1 phenotype and promoting DC maturation.^325^ Treatment with IL21-armed OVV was shown to enhance TIL activity and showed notable synergy with CAR T-cell therapy in tumor treatment.^234^ In another study, an engineered OAd loaded with IL-7 was used in combination with B7H3-targeted CAR T cells, and this combination treatment enhanced T-cell proliferation, reduced the T-cell apoptosis rate and improved the therapeutic efficacy of B7H3-CAR T cells in glioblastoma.^326^ Similarly, a combination of armed OVs with CAR NK cells achieved a profound therapeutic effect. Ma et al. constructed an oncolytic HSV-1 to express the human IL15/IL15Rα complex (named OV-IL15C) to investigate its efficacy when administered with EGFR-CAR NK cells in multiple glioblastoma mouse models.^327^ Compared with monotherapy, the combination therapy increased intracranial infiltration and activation of NK and CD8^+^ T cells and prolonged the persistence of CAR NK cells, leading to tumor growth inhibition and prolonged survival of tumor-bearing mice.^327^

TGFβ plays a critical role in T-cell exclusion and immunosuppressive microenvironment formation.^328^ Targeting TGFβ activity has demonstrated promise and efficacy in tumor therapy.^329^ Soluble TGFβ receptor II fusion protein (sTGFβRIIFc), a TGFβ antagonist, has been demonstrated to suppress metastasis in mice.^330^ Combining the effect of OV oncolytic activity on tumor cells and the function of sTGFβRIIFc to block TGFβ signaling, OAd expressing sTGFbRIIFc (Ad.sTbRFc) significantly inhibited breast cancer metastasis in mice.^331,332^ Furthermore, Li et al. combined Ad.sTbRFc with mesothelin-targeted CAR T cells to develop a better therapeutic strategy.^333^ According to the results of their study, Ad.sTbRFc obviously inhibited tumor growth at the early stage of treatment. In contrast, mesothelin CAR T cells showed greater antitumor responses at a later stage. The combined therapy mediated a stronger long-term antitumor response than monotherapy.^333^

### OVs overcome antigen loss or diversity

Identifying and clearing tumor cells by CAR T and TCR T cells require that target antigens are presented on cells. CAR T cells recognize tumor antigens on the cell surface; in contrast, TCR T cells target intracellular antigens or cell surface antigens. Antigens exclusively presented on tumor cells but not healthy cells are prerequisites for safe and effective CAR T and TCR T-cell therapy for solid cancers.^334^

However, solid tumors are in an immunosuppressive TME characterized by heterogeneous antigens and lack of targetable tumor antigens, creating a challenge to the effective clinical use of CAR T and TCR T-cell therapeutics.^335^ Considerable effort has been devoted to developing ACT strategies for overcoming antigen heterogeneity in solid tumors.^336^

OVs combined with bispecific T-cell engagers (BiTEs) target various antigens and overcome antigen escape during ACT. For example, OAd delivering an EGFR-targeting BiTE (OAd-BiTE) was used to improve the efficacy of folate receptor alpha (FR-α)-specific CAR T-cell therapy by overcoming the problem of tumor heterogeneity in solid tumors.^337,338^ The cytotoxicity of FR-α-targeted CAR T cells is closely associated with FR-α density. FR-α-negative cancer cells can escape recognition and killing by CAR T cells. However, BiTEs expressed by OAd-infected cells effectively redirected CAR T cells toward EGFR-positive and FR-α-negative cancer cells, resulting in a reduction in tumor heterogeneity, improved antitumor efficacy and prolonged survival in mouse models of cancer.^337^ Furthermore, Suzuki and collaborators constructed an OV that produced IL-12, an anti-PD-L1 antibody, and a CD44v6-targeted BiTE molecule (forming CAdTrio), enhancing the breadth, potency, and duration of the antitumor activity of HER2-specific CAR T cells.^339^ CD44v6 BiTEs secreted from CAdTrio redirected HER2-specific CAR T cells to kill CD44v6-positive cancer cells and induce dual targeting of orthotopic HER2^+^ and HER2^−/−^ CD44v6^+^ tumors.^339^ Based on the confirmed capability of BiTEs, bi and tri specific T-cell engager-armed OVs might be promising in tumor treatment.^340^

Recently, local intratumoral delivery of antigens by OVs improved CAR T-cell immunotherapy and demonstrated remarkable efficacy with nonimmunogenic solid tumors. Park et al. engineered an OVV to generate a nonsignaling truncated CD19 protein (CD19t) that was a B-cell-lineage-restricted molecule.^341^ Infected with this armed OV, CD19-negative triple-negative breast and glioma tumor cells specifically expressed CD19t residing on the cell surface. When these cells were cocultured with CD19-targeted CAR T cells in vitro, the cytotoxicity of the T cells was significantly increased, as indicated by the upregulated expression of activation markers (CD25 and CD137), increased levels of secreted cytokines IFN-γ and IL-2, and lysis of CD19^+^ tumor cells. This combination therapy resulted in remarkable tumor regression compared to monotherapy in immunodeficient NSG mice. Additionally, the authors demonstrated that OV19t promoted endogenous T-cell and CAR T-cell infiltration into tumors and induced immunological memory in immunocompetent mouse tumor models.^341^ Furthermore, Aalipour et al. confirmed the efficacy of OVs as carriers to induce targets of CAR T cells.^342^ However, some strategies are focused on delivering CAR T targets into tumors. For example, CAR T cells engineered to coexpress antigen peptides can transfer antigen peptides to tumor cells via extracellular vesicles, improving the presentation and targeting by antigen-specific CTLs for the treatment of nonimmunogenic tumors.^343^ In another study, recombinant AdV was used to deliver truncated CD19 tags into a number of cancer cell lines to improve CD19 CAR T-cell therapeutic efficacy, overcoming the problem of endogenous antigen dependence.^344^ In summary, the multiple advantages of a tumor-tagging strategy combining OVs with CAR T cells make this combination a novel and promising solution for the heterogeneity and antigen loss in solid tumors.^345^

### OVs attenuate exhaustion of CAR T and TCR T cells

Exhaustion and senescence, two crucial dysfunctional states of T cells in the TME, limit the efficacy and application of ACT.^346^ Exhausted CAR T cells are dysfunctional, and this state is acquired mainly through the upregulation of multiple inhibitory receptors, such as PD-1, CTLA-4, and TIM-3.^347^ High expression of PD-1 has been observed in TCR T cells following infusion, and this expression was associated with reduced production of IFN-γ and a decreased immune response.^348^

Hence, disrupting the PD-1/PD-L1 axis is an effective way to relieve adoptive T-cell exhaustion and improve persistence. An anti-PD-1 blockade antibody was used to enhance the function of CAR T or TCR T cells and thereby promote tumor eradication,^349,350^ but this approach might lead to the development of systemic toxicity.^351^ Self-delivery of PD-1 blocking ScFv via engineered CAR T cells is a safe strategy to augment these cell functions and persistence in the TME.^352^ Local secretion of functional checkpoint blockade factors by armed OVs may be a simple, safe and efficacious approach to boost the efficacy of CAR T cells. For example, Suzuki and colleagues engineered OAV to express an anti-PD-L1-blocking mini-antibody (CAd-VECPDL1) to enhance CAR T-cell killing action.^353^ This anti-PD-L1 mini antibody was detected at the tumor site after CAd-VECPDL1 administration. The combination of CAd-VECPDL1 with HER2-targeted CAR T cells showed enhanced antitumor activity compared to treatment with HER2-targeted CAR T cells alone, HER2-targeted CAR T cells plus unarmed OAd and even anti-PD-L1 blocking antibody plus HER2-targeted CAR T cells in a HER2 prostate cancer xenograft model. These data demonstrated the superiority of the local production of anti-PD-L1 mini antibodies by OVs in combination with ACT.^353^

### OVs enhance the adoptive transfer of TILs

Immunotherapy using autologous TILs is an adoptive cell transfer therapy and has emerged as a powerful treatment option for patients with advanced solid tumors, especially metastatic melanoma.^354^ TIL therapy refers to the surgical excision of tumors from patients, isolation and expansion of TILs ex vivo and then the transfer of TILs back into the same patient.^355^ Adoptive transfer of TILs for the treatment of metastatic melanoma has shown high efficacy, with objective responses ranging from 40% to 70%,^356^ which were largely associated with the high mutational load and abundant tumor-reactive lymphocytes in the tumors. However, most solid tumors are poorly immunogenic, and tumor tissues lack TILs, which become major hurdles in sourcing TILs.^357^ Unarmed or armed OV-mediated ICD causes the release of TAAs/TANs and can turn immunologically “cold” tumors into “hot” tumors accompanied by TIL accumulation, which makes OVs great partners for TIL therapy. Recently, one study reported a combination of OV and TIL therapy. Feist et al. intratumorally injected IL2-armed oncolytic poxvirus into MC38 tumors with low immunogenicity, and the results showed the accumulation of tumor-specific TILs that contained a lower percentage of exhausted PD-1^hi^Tim-3^+^CD8^+^ T cells and Tregs. TILs, undergoing isolation, expansion and transfer, significantly delayed the growth of tumors and improved the survival of mice with established MC38 tumors.^288^

## Outlook

OVs can selectively kill tumor cells, but first-generation OVs (wild-type and natural variant strains of weak viruses) have low clinical activity. Between first-generation OVs and third-generation OVs (exogenous therapeutic gene-“armed” OVs), a great deal of effort has been directed to understanding the activating effect of OVs on antitumor immunity. As OVs can turn “cold” tumors into “hot” tumors and can be readily genetically engineered with immunomodulatory therapeutic genes, it is possible and promising to use these OVs as platforms to enhance T-cell function against tumors. For currently used cytokines and ICIs expressed by OVs, more effective drug targets will certainly be found in the near future. To date, the combination of OVs with ICIs or ACT to promote a sustained antitumoral immune response has been successfully tested in preclinical studies and in clinical trials. Further efforts should be directed to realize oncolytic monotherapy or combinations of OVs with other immunotherapies in cancer treatment to improve T-cell responses.
